# Deciphering the genetic landscape of obesity: a data-driven approach to identifying plausible causal genes and therapeutic targets

**DOI:** 10.1038/s10038-023-01189-3

**Published:** 2023-08-24

**Authors:** Mia Yang Ang, Fumihiko Takeuchi, Norihiro Kato

**Affiliations:** 1 Department of Clinical Genome Informatics, Graduate School of Medicine, The University of Tokyo, Tokyo, Japan; 2 Department of Gene Diagnostics and Therapeutics, Medical Genomics Center, Research Institute, National Center for Global Health and Medicine, Tokyo, Japan

**Keywords:** Gene regulatory networks, Data mining, Data processing

## Abstract

**Objectives:**

Genome-wide association studies (GWAS) have successfully revealed numerous susceptibility loci for obesity. However, identifying the causal genes, pathways, and tissues/cell types responsible for these associations remains a challenge, and standardized analysis workflows are lacking. Additionally, due to limited treatment options for obesity, there is a need for the development of new pharmacological therapies. This study aimed to address these issues by performing step-wise utilization of knowledgebase for gene prioritization and assessing the potential relevance of key obesity genes as therapeutic targets.

**Methods and results:**

First, we generated a list of 28,787 obesity-associated SNPs from the publicly available GWAS dataset (approximately 800,000 individuals in the GIANT meta-analysis). Then, we prioritized 1372 genes with significant in silico evidence against genomic and transcriptomic data, including transcriptionally regulated genes in the brain from transcriptome-wide association studies. In further narrowing down the gene list, we selected key genes, which we found to be useful for the discovery of potential drug seeds as demonstrated in lipid GWAS separately. We thus identified 74 key genes for obesity, which are highly interconnected and enriched in several biological processes that contribute to obesity, including energy expenditure and homeostasis. Of 74 key genes, 37 had not been reported for the pathophysiology of obesity. Finally, by drug-gene interaction analysis, we detected 23 (of 74) key genes that are potential targets for 78 approved and marketed drugs.

**Conclusions:**

Our results provide valuable insights into new treatment options for obesity through a data-driven approach that integrates multiple up-to-date knowledgebases.

## Introduction

Obesity is a multifaceted condition characterized by excessive fat accumulation in the body, often associated with chronic conditions such as heart disease, diabetes, high blood pressure, and cancers [1]. Despite concerted efforts, the prevalence of obesity has significantly increased, with the proportion of obese adults in the United States rising from 30.5% to 42.4% in less than two decades [2]. While lifestyle modifications have limited efficacy in controlling obesity, few available drugs are serving as anti-obesity agents [3]. Unfortunately, current research methods are insufficient for developing personalized therapies, and the traditional drug discovery process is time-consuming, laborious, expensive, and risky [4]. Furthermore, concerns exist regarding the long-term effects of FDA and EMA-approved weight-loss drugs [5]. Therefore, it is imperative to address obesity seriously, necessitates effective strategies to identify and target associated key genes and pathways.

Genome-wide association studies (GWAS) represent significant advancement in sequencing technology for identifying genetic associations with various traits and diseases. Nevertheless, GWAS encounters several inherent limitations [6], including non-coding variants introducing complexity and necessitating tissue-specific exploration contexts, as well as the proximity of closely situated genes, which complicates the determination of their significance. Furthermore, linkage disequilibrium (LD) can result in false positives, obscuring the identification of true causal variants. Additionally, complex diseases often arise from disruptions in intracellular biological network, rather than single gene abnormalities.

Despite considerable efforts to investigate the functional implications of obesity-related GWAS [7, 8], certain gaps persist. Previous research has investigated the genetic regulation of blood pressure regulatory genes using post-GWAS data [9], but similar investigations for obesity remain limited. Although potential causal SNPs and hub genes [10] have been identified based on their proximity to GWAS signals, the lack of eQTL data and investigation of relevant tissues has hindered causal inference. Additionally, drug repositioning application in the post-GWAS analysis of obesity have not been addressed. Nonetheless, a recent study employed expression datasets to identify differentially expressed genes and screened potential drugs targeting important obesity hub genes [11].

**Fig. 1:**
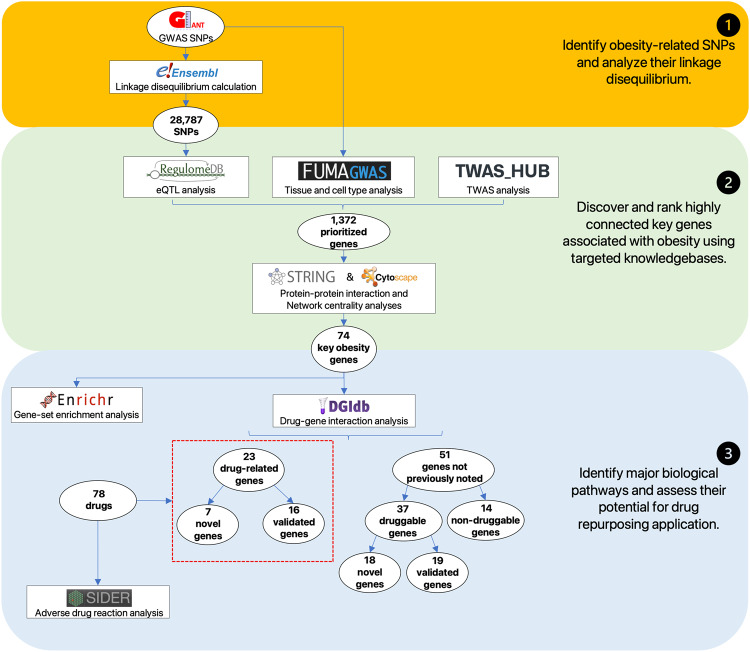
Overview of the data-driven integrative approach. We extract 28,787 obesity-associated SNPs from publicly available GWAS results (top panel) and systematically prioritize 74 plausible key obesity genes, by utilizing a series of bioinformatics tools and genomic and transcriptomic evidence (middle panel). We then explore major biological mechanisms of obesity from the key obesity genes, highlighting 23 potential candidates that are useful for the development of obesity therapeutics (low panel)

Accordingly, we conducted data-driven integrative analysis by leveraging a credible GWAS dataset [8] with updated bioinformatics tools and knowledgebases [12–14], prioritizing obesity-associated genes with significant in silico evidence. Expanding upon previous research [7], our study incorporated a larger study population, allowing us to identify and update the most plausible causal genes and evaluate their clinical relevance as potential therapeutic targets. Protein-protein interaction [15] and network centrality analysis [16] pinpointed key genes, while gene-set enrichment analysis [17] shed light on underlying biological processes and pathways. Drug-gene interactions [18] analysis, as well as adverse drug reactions [19], unveiled promising opportunities for drug repurposing. Our study informs future obesity research and guides future experimental assays to investigate mechanisms and targeted therapies. An overview of this study is illustrated in Fig. 1.

## Materials and methods

### GWAS SNPs analysis

Associations identified through GWAS provide a foundation for investigating the biological underpinning of obesity. Given this, we compiled a credible catalog of 941 near-independent genome-wide significant SNPs (COJO P < 1E-08), which captured meaningful association in the GWAS while minimizing potential false positives [20], identified from the Genetic Investigation of Anthropometric Traits (GIANT) consortium, the largest centralized BMI GWAS dataset derived from ~800,000 individuals [8]. To identify additional SNPs that equally contribute to obesity, we calculated linkage disequilibrium (LD) using genotype data from the 1000 Genomes Phase 3 Project, focusing on the European ancestry (CEU) population reference panel. Our search criteria included a distance range of ± 500 kb from the query significant SNPs and *r*^*2*^ > 0.9 from pairwise LD calculation. These LD SNPs were consolidated together with the genome-wide significant SNPs to create a list of obesity-associated SNPs.

### eQTL analysis

Integrating expression Quantitative Trait Loci (eQTL) data with GWAS offers insights into the genetic variance associated with changes in gene expression of disease phenotypes [21]. To prioritize eQTL-genes and explore their potential regulatory roles, we utilized RegulomeDB [12], a comprehensive knowledgebase that provides functional interpretation of SNPs based on curated references, where these SNPs were scored based on their combinatorial existence of functional categories. We focused specifically on category 1 variants (1a–1f), also known as eQTL-SNPs which demonstrate strong evidence of influencing the expression of eQTL-genes associated with obesity.

### Tissue expression analysis

Tissue expression analysis revealed specific tissues where particular genes are expressed, highlighting their potential involvement in disease pathogenesis [22]. To determine specific tissues associated with obesity, we performed tissue expression analysis using FUMA, a web platform capable of performing functional mapping and annotation of genetic variations identified in GWAS studies. Within FUMA, we utilized Multi-marker Analysis of GenoMic Annotation (MAGMA) [23] to evaluate the enrichment of genes in specific tissues and prioritized differentially upregulated genes for further functional analysis of their potential roles in the pathophysiology of obesity.

### TWAS analysis

Transcriptome-wide association studies (TWAS) integrate genotype and phenotype data from GWAS with reference expression panels, providing insights into potential causal genes in diseases [24]. TWAS complements GWAS findings by uncovering genes that are missed by GWAS, providing additional regulatory evidence. Using TWAS Hub [14] with searchable access to TWAS results of complex traits and expression studies, we identified gene expression associated with obesity-related phenotypes, including BMI, fat mass, waist circumference, and weight. Considering the emerging role of the brain in weight regulation [25], we focused on transcriptionally regulated genes associated with the brain, such as caudate basal ganglia, cerebellar hemisphere, cerebellum, cortex, frontal cortex BA9, hippocampus, hypothalamus, nucleus accumbens basal ganglia and putamen basal ganglia. These genes were prioritized for further investigation.

### Protein-protein interactions and network centrality analysis

Protein-protein interactions (PPI) play a crucial role in regulating biological processes and provide valuable insights into the functions and interactions of proteins within the cells [26]. To better understand the network involved in obesity-associated genes, we used STRING [15] to reconstruct networks by integrating associations between proteins derived from computational approaches and assigning confidence scores (0-1) to quantify the strength of supporting evidence. We chose the default medium confidence score of 0.4 to interpret the interactions between the obesity-associated genes. Given the significance of network centrality analysis in identifying important hub genes [27], we employed cytoHubba [16] to perform a topological analysis of the network structure using centrality algorithms such as betweenness, closeness, and degree to assess the importance of individual proteins within the network. We ranked the top 100 hub genes using these algorithms and identified key genes by finding overlaps in the resulting lists.

### Gene set enrichment analysis

Gene Set Enrichment Analysis (GSEA) integrates knowledge about the function of a group of genes, taking into account their involvement in specific biological pathways or co-expression under certain conditions [28]. Gene ontology (GO) is commonly used for gene functional annotation covering biological processes, molecular functions, and cellular components. Similarly, the Kyoto Encyclopedia of Genes and Genomes (KEGG) increases the explanatory power of specific gene sets to gain insights into underlying biology pathways. To identify significantly enriched GO and KEGG terms of the key obesity genes, we utilized Enrichr [17], an integrative web-based application. Enriched GO and KEGG pathways terms with adjusted *p*-value < 0.05 and involving > 3 genes were considered statistically significant.

### Drug-gene interaction and adverse drug reaction analysis

Drug-gene interaction (DGI) refers to the interaction between genes and drugs that can potentially influence drug responses [29]. To identify target genes and potential drugs that interact with key obesity genes, we queried the Drug Gene Interaction Database (DGIdb) [18], a consolidated resource of DGI interactions and druggable genes. We focused on FDA-approved drugs supported by evidence from two or more databases and PubMed sources, considering them as potential candidates for drug repurposing in obesity. Adverse drug reaction (ADR) refers to unintended and potentially harmful effects arising from the therapeutic use of medications [30]. To examine the safety profile of our repurposed drug candidates, we conducted a search using the Side Effect Resource (SIDER) database [19], a resource containing information on potential side effects of approved drugs. Specifically, we focused on side effects classified as “very common” according to the Medical Dictionary for Regulatory Activities (MedDRA) hierarchy, as they have a frequency of ≥ 10% and are likely to occur in a significant proportion of patients using the medication.

### Negative control and benchmarking analysis

Negative control and benchmarking analysis are crucial for experimental reliability [31]. In our workflow, we first prioritized obesity-associated genes by integrating multiple human sources. In the second part, we identified highly interconnected hub genes in the PPI network. To validate the effectiveness of each step, we conducted analyses with negative controls, resulting in different sets of key genes. We then calculated the overlap of these genes with genes from the mouse knockout database [32] and drug-related information from the drug-gene interaction database [18]. Additionally, we performed two benchmarking analyses by reanalyzing previous investigations of BMI GWAS study [7], utilizing GWAS summary statistics and prioritized genes as starting materials, respectively.

## Results

### Identification of obesity-associated SNPs

To obtain a credible list of genetic associations with obesity, we acquired 941 near-independent genome-wide significant SNPs from BMI GWAS of the GIANT consortium, and subsequently examined representative signals of obesity based on LD. From the significant SNPs, we identified 27,846 LD SNPs applying a threshold of ± 500 kb from the query SNPs and an *r*^2^ > 0.9. After trimming off overlapping SNPs, a non-redundant list of 28,787 obesity-associated SNPs was assembled, which were then clustered into 640 genomic loci based on < 500 kb distances (Supplementary Table 1).

### Prioritization of obesity eQTL-SNPs and eQTL-genes

To identify SNPs located in regulatory regions of the genome, we employed RegulomeDB to score the obesity-associated SNPs for regulatory functions. Of the 28,787 SNPs examined, 25,776 ( ~ 90%) were assigned with putative regulatory functions. From these, we extracted 867 putative eQTL-SNPs exhibiting high potential for regulatory function (score 1) for downstream analysis. These SNPs were predicted to have an influence on the expression of 243 eQTL-genes, which were prioritized based on their potential to cause obesity through changes in gene expression (Supplementary Table 1).

### Prioritization of differentially upregulated genes in obesity tissues

To identify the most relevant tissues associated with obesity, we used MAGMA [23], which was incorporated in FUMA [13] for tissue expression analysis. Our findings revealed that nearly all brain tissues (10 out of 13) were significant at *P* < 0.001, with the brain cerebellum having the strongest *p-value* (*P* = 5.45 × 10^−^^15^), demonstrating a strong relationship between the brain and obesity. Additionally, pituitary tissue was also significant with a *P* = 6.48 × 10^−^^5^. Subsequent differential analysis identified a set of differentially upregulated genes found in these brain tissues at adjusted *P* < 0.05 (Supplementary Table 2). Notably, cortex exhibited the strongest upregulation pattern (*P* = 3.25 × 10^−^^6^), followed by frontal cortex BA9 (*P* = 8.04 × 10^−^^6^), and cerebellum (*P* = 1.17 × 10^−^^5^), with slightly weaker but still strongly significant upregulation observed in the cerebellar hemisphere (*P* = 1.28 × 10^−^^4^) and anterior cingulate cortex BA24 (*P* = 3.05 × 10^−^^4^). A total of 845 differentially upregulated genes from these brain tissues were prioritized for downstream analysis on their potential roles in causing obesity.

### Prioritization of TWAS genes associated with obesity

TWAS enabled the identification of genes whose expression is associated with obesity. We analyzed TWAS experiments from TWAS Hub [14], specifically targeting 4 phenotypes capable of defining obesity. Given the enrichment of brain tissues in our tissue enrichment analysis, we expanded our analysis to include TWAS experiments to uncover additional genes involved in transcriptional activity related to obesity. We prioritized 396 genes exhibiting transcriptional activity in the brain, which are listed in Supplementary Table 3. A total of 63 genes are shared between both TWAS Hub and FUMA. In addition, 31 genes were found to be common between TWAS Hub and RegulomeDB, with a total of 14 genes identified in all three targeted knowledge resources.

### Identification of key obesity genes

We aimed to explore the interconnectedness of genes implicated in obesity, deducing their significance in important biological pathways associated with obesity by being closely integrated within a protein network. To achieve this, we merged the genes prioritized based on in silico evidence from targeted knowledge resources, namely RegulomeDB (243 genes), FUMA (845 genes), and TWAS Hub (396 genes), resulting in a gene set of 1,372 genes associated with obesity.

**Fig. 2:**
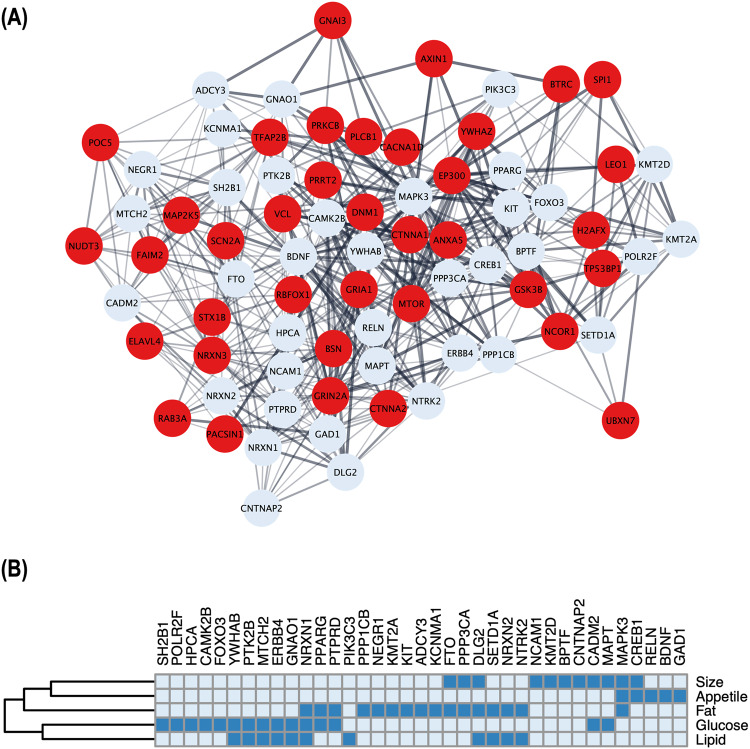
Relationship and classification of key obesity genes identified through network analyses. **A** Schematic illustration of a protein-protein interaction (PPI) subset involving 74 key obesity genes, where thicker edges indicate stronger data support. Of these, 37 red nodes represent newly reported genes that have not been functionally validated for obesity. **B** For the 37 functionally validated known genes, the heatmap shows their involvement in five phenotypic groups i.e., appetite, fat, size, lipid, and glucose, reported in the literature; presence by dark blue and absence by light blue

To identify potential physical and functional associations among these genes, we utilized STRING [15] and applied a minimum interaction score > 0.4. Subsequently, we employed cytoHubba [16] to evaluate the nodes in the PPI network using three centrality parameters, namely betweenness, closeness, and degree, and ranked the top 100 hub genes from each algorithm respectively. By selecting genes that overlapped in all three network centrality analyses, we identified 74 key obesity genes highly integrated within a protein network (Fig. 2A). Through extensive literature review, which included assessing single gene knock-out experiments conducted by the International Mouse Phenotyping Consortium (IMPC), we classified these key genes into predicted novel (*n* = 37) and functionally validated (*n* = 37). For the functionally validated known genes, we further categorized them to their association with obesity, based on their regulation of appetite, fat, size, lipid, and glucose (Fig. 2B).

### Identification of major biological pathways associated with obesity

**Fig. 3:**
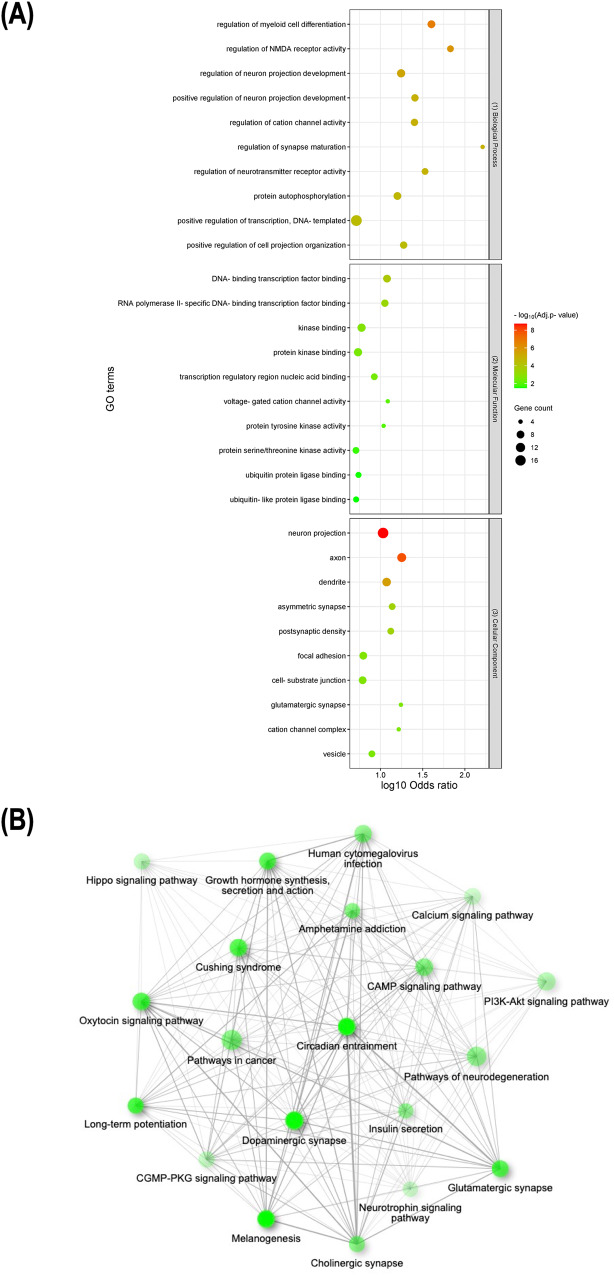
Representative results for enrichment analyses of key obesity genes. **A** Lists of the top 10 significantly enriched GO terms from biological processes (top), molecular functions (middle), and cellular components (low), respectively. **B** Schematic illustration of pairwise relationships between top 20 significantly enriched KEGG pathways, where darker and larger nodes indicate more significantly enriched and larger gene sets and thicker edges represent more overlapped genes

To further investigate the biological significance of the key obesity genes, we performed gene set enrichment analysis using Enrichr. To indicate strong enrichment, stringent parameters such as adjusted *p*-value < 0.05 and the presence of more than 3 genes in a gene set were employed (Fig. 3). Our analysis indicated that the key obesity genes were significantly enriched in a total of 119 GO terms, with 91 terms (~76%) associated with biological processes, 12 terms (~11%) associated with molecular functions, and 16 terms (~13%) associated with cellular components (Supplementary Table 5). In addition, 104 KEGG pathway terms were significantly enriched (Supplementary Table 6).

### Identification of repurposed drug candidates and adverse drug reactions

**Fig. 4:**
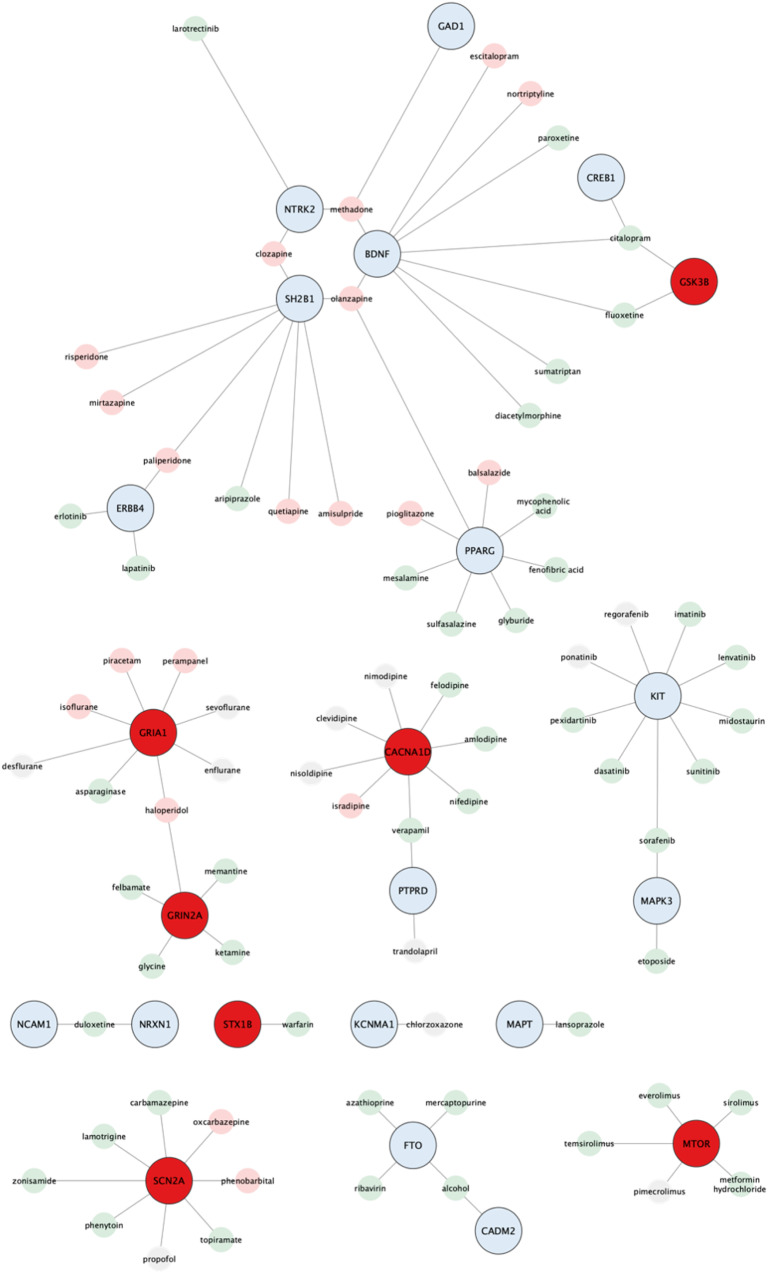
Schematic illustration of 23 key obesity genes and 78 FDA-approved drugs. Genes highlighted in blue are validated, while those in red are not for functional relevance to obesity. These drugs are further classified into two groups based on their experimental evidence; promotion of weight gain (pink) and weight loss (light green) in case of over-expression of the corresponding gene product

Key genes are essential in maintaining the structure and function of PPI networks, making them attractive candidates for novel therapeutics. In search of potential therapeutic targets, we utilized DGIdb to analyze drug-gene interactions among the key obesity genes. We focused on reliable interactions supported by at least two resources and PubMed references while excluding cancer-specific resources and limiting our search to FDA-approved drugs or drugs in clinical trials (Fig. 4). Among the 74 key obesity genes, we identified 23 drug-related genes (Supplementary Table 7), as well as 51 genes not previously noted. Further analysis of the genes not previously noted showed 37 druggable and 14 non-druggable (Supplementary Table 8). The 23 drug-related genes were found interacting with 78 drugs, where of these drugs, 47 drugs can lead to weight loss, another 19 were associated with weight gain, while 12 had unknown effects based on prior reports.

To evaluate the safety profile, we searched the SIDER database for side effect data of the 78 drug candidates, focusing on side effects classified as “very common” to identify the most frequent occurrences. Among the 78 drug candidates, 19 had reported side effects (Supplementary Fig. 1). These findings could inform future research on the safety and potential use of these drugs for obesity management in clinical settings (Supplementary Table 9).

### Validation of workflow through negative control and benchmarking analyses

Our workflow underwent rigorous validation tests to ensure its reliability. It involved two steps: (1) prioritizing 1372 obesity-associated genes by integrating multiple human data sources, and (2) narrowing down the list to 74 key hub genes in the PPI network. The mice knockout database [32] and drug-gene interaction database [18] both revealed 21 genes (~28%) with reported mice knockout abnormalities and 23 drug-related genes (~31%).

**Fig. 5:**
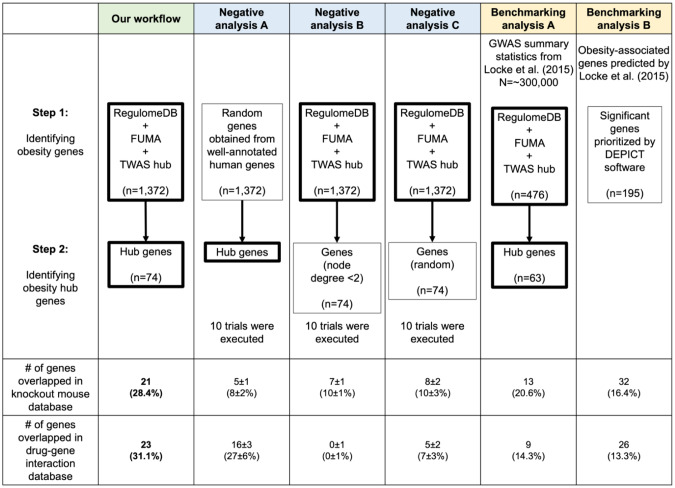
Effectiveness of workflow against negative-control and benchmarking analyses. We evaluate the effectiveness of our workflow by comparison with negative control and benchmarking analyses. Genes overlapping between the mice knockout database and the drug-gene interaction database are counted. The thick border represents our method, with 10 trials conducted for negative control analyses. The number and percentage of genes that overlapped in negative control analyses are shown as median and standard deviation

To evaluate the effectiveness of the first step, we randomly selected 1372 genes from a pool of 14,937 well-annotated human genes (obtained from Enrichr’s GO Biological Process library) and performed hub genes identification analysis using our workflow ten times (Fig. 5). Our workflow detected a significantly greater percentage of genes with mice knockout abnormalities compared to the negative analyses, indicating effective enrichment of true obesity genes (t-test *P* < 0.00001). Additionally, the percentage of genes with drug-related information was also significantly greater at *P* = 0.035517. To evaluate the second step, we randomly selected two groups of 74 genes from the 1372 prioritized obesity-associated gene set to serve as substitutes for hub genes and repeated the analysis ten times. The first set comprised genes with limited connections in the PPI network (node degree < 2), while the second set consisted of genes without considering their interconnectivity information. Our analysis revealed a significantly greater percentage of genes with drug information in our workflow than the negative analyses (t-test *P* < 0.00001), indicating effective enrichment of true drug target genes. Moreover, genes with lower node degree had a lower likelihood of having drug-related information compared to genes with higher node degree.

Lastly, leveraging the GWAS summary statistics and 195 significant genes prioritized from a previous investigation of BMI GWAS [7], we conducted two benchmarking analyses. Our workflow outperformed both benchmarking analyses in identifying a higher percentage of genes associated with mice knockout abnormalities and drug information. This improvement can be attributed to the utilization of a larger GWAS size (Benchmarking analysis A) and updated bioinformatics tools (Benchmarking analysis B) as demonstrated in Supplementary Table 10. Our findings showed the importance of incorporating larger sample sizes and employing up-to-date bioinformatics tools are crucial for identifying key genes and druggable targets.

## Discussions

In this study, we prioritized obesity genes with potential causal roles utilizing targeted knowledgebases and identified relevant biological processes and pathways for clinical translation in obesity drug applications. Leveraging GWAS with a larger sample size, we conducted a series of integrative analyses using the latest bioinformatics tools and knowledgebases. Exploring the relationship between SNPs and gene expression, we prioritized 243 eQTL-genes. We then examined tissue expression and prioritized 845 differentially upregulated genes from the brain and another 396 transcriptionally regulated genes from TWAS experiments, highlighting expression patterns of the brain in obesity. Establishing a ~1400 prioritized obesity-associated gene set, we performed protein-protein interaction analysis and identified 74 key genes enriched in biological pathways regulating feeding behavior, energy expenditure, metabolic homeostasis, and insulin secretion. Lastly, our drug-gene interaction analysis identified 23 genes targeted by 78 existing drugs in repurposing applications, as well as additional 37 druggable genes with potential for drug development, offering valuable insights for new obesity therapeutics. Our workflow was inspected through negative and benchmarking analyses, where a greater percentage of genes associated with mice knockout abnormalities and drug-related information were identified, indicating its effectiveness.

Our study presents two novel findings that contribute to the current understanding of genetic regulation and therapeutic options for obesity. The first finding addresses the lack of knowledge regarding regulatory genes involved in obesity pathogenesis. While comprehensive integrative analysis with updated bioinformatics tools and knowledgebases on post-GWAS data of blood pressure [9], the field of obesity has lagged behind in achieving similar advancements, despite recent computational analysis of obesity-associated GWAS SNPs [10] that focused on genes closest to the GWAS signals. To fill this gap, we implemented a data-driven annotation strategy that employed three-layer evidence from targeted knowledgebases to prioritize regulatory genes associated with obesity. Our analysis prioritized 74 key genes associated with obesity, half of which were predicted novel genes that lacked functional validation in relation to obesity. The second novel finding concerns drug repurposing for obesity treatment. While previous studies [11] have identified drugs using expression datasets, no attempt has been made using post-GWAS obesity data. Our approach identified 23 drug-related genes and 78 drugs supported by evidence from human and animal experiments suitable for repurposing as novel therapeutic options for obesity. On the remaining 51 genes not previously noted, 37 were found to be druggable, providing opportunities for future drug development. To ensure the validity of our findings, negative analyses were performed at every major step of our analysis and demonstrated the significant improvement of our prediction workflow compared to previous reports, predicting and identifying truly enriched key obesity genes, and potential drug target genes for obesity.

In contrast to a previous study [10] that focused on the nearest genes to obesity-associated SNPs, our study instead highlights the relevance of studying eQTL-genes in explaining the regulatory mechanisms of obesity. To investigate this, we gathered significant GWAS SNPs from BMI GWAS of the GIANT consortium and calculated LD to find SNPs contributing equally to obesity. We identified 867 eQTL-SNPs with a RegulomeDB score of ≤ 1, capable of regulating a total of 243 eQTL-genes associated with obesity. Among these, 63 of them were genome-wide significant eQTL-SNPs capable of regulating 76 eQTL-genes. Tissue expression analysis enables the identification of genes expressed in particular tissues, providing insights into their contribution to the various cell types and organs. To supplement the prioritized eQTL-genes that lack tissue-specific information, we conducted tissue expression analysis and prioritized 845 differentially upregulated genes present in the brain tissues, consistent with previous studies from other groups proposing that brain tissues play a crucial role in the regulation of body weight and the development of obesity [7, 33].

Emerging evidence suggests that vulnerability to obesity extends across multiple brain regions, receiving signals from internal and external sources to collectively regulate feeding habits and energy storage [34, 35]. Also, a previous study [36] reported the enrichment of neuronal cells in the brain, providing valuable evidence of the neurological consequences of obesity. The brain is extensively reviewed as a critical regulator of metabolic traits and physiological processes, including energy metabolism and glucose regulation in the central nervous system [37]. Moreover, another study showed that the dysregulation of neuronal pathways in the brain disrupts energy balance, resulting in excessive food intake and reduced energy expenditure in mice [38]. Enrichment in brain tissues prompted us to expand our investigation by integrating TWAS experiments to identify and prioritize an additional 396 transcriptionally regulated genes associated with the brain, 63 of which were previously identified in our tissue expression analysis.

PPI provides valuable insights into the organization and coordination of biological processes underlying diseases. Specifically, our analysis identified 74 key genes, of which 37 were predicted novel and have not been functionally validated in relation to obesity. We further examined the potential association of these novel genes to shed light on their underlying mechanism. *ANXA5* is known for anticoagulation and is involved in triglyceride biosynthesis [39]. Additionally, *AXIN1 and BTRC* regulate adipose tissue lipogenesis through the Wnt signaling pathway [40]. *CACNA1D* affects insulin secretion and has been linked to various conditions [41]. Other genes, such as *CTNNA1* and *CTNNA2* are involved in the Hippo signaling pathway, regulating adipogenesis [42]. Impairment of *DNM1* causes insulin secretion failure and hyperglycemia in mice [43] while inhibiting the expression of *EP300* reduces adiposity in larval zebrafish [44]. *FAIM2* is linked to obesity and dyslipidemia in the Chinese population [45], while *GNAI3* is associated with non-alcoholic fatty liver disease (NAFLD) [46]. Furthermore, *GRIA1* influences appetite in T2D patients [47]. *GSK3B* regulates inflammation in diabetes patients [48]. *MAP2K5* is known to activate *ERK5*, a critical regulator of adipogenesis through the PKA signaling pathway [49], while inhibiting *MTOR* signaling resulting in reduced food intake and body weight in mice [50]. The homolog of *NUDT3* in Drosophila, *Aps*, is involved in insulin signaling [51], while defects in *PACSIN1* have been associated with schizophrenia-like behavior in mice, another condition linked to obesity [52]. *PRKCB* deficiency reduces the obesity syndrome of mice [53], while *RAB3A* is involved in the regulation of insulin secretion [54]. Moreover, *RBFOX1* regulates *BDNF*, crucial for neuronal development and energy metabolism in mice [55]. *TFAP2B* is linked to insulin resistance and adiposity [56]. These predicted novel genes are involved in various biological pathways, such as lipid and energy metabolism, insulin secretion, adipogenesis, and neural development. Further investigation is required to evaluate the potential link between *BSN, GRIN2A, H2AFX, LEO1, NCOR1, NRXN3, PLCB1, POC5, PRRT2, SCN2A, SPI1, STX1B, UBXN7, VCL, YWHAZ*, and obesity.

Subsequently, we examined the 74 key obesity genes to determine their presence in prior analysis by Locke’s GWAS dataset [7], as reported by DEPICT software [57]. Among these 74 genes, DEPICT identified 36 genes, while the remaining 38 genes were not reported. Notably, within this group of unreported genes, we observed that 12 genes were situated close to the sentinel SNPs of Yengo’s GWAS dataset [8] (dataset used in this analysis), with distances ranging from 0 (closest) to 18,724 base pairs (farthest). Interestingly, only one gene among these 12 genes was also found to be situated near the sentinel SNPs of Locke’s GWAS datasets [7]. Conversely, the remaining 26 unreported genes were not found to be the nearest genes to the GWAS SNPs. These findings highlight the importance of larger GWAS datasets and updated bioinformatics tools in achieving greater precision in research outcomes.

GO terms and KEGG pathways enrichment analysis offers valuable conclusions about gene sets. Our GO analysis indicated that key obesity genes are enriched in biological processes related to the brain and nervous system, such as myeloid cell differentiation, NMDA receptor activity, and neuron projection development. Deficiencies in myeloid cells protect mice from diet-induced obesity and insulin resistance [58], while NMDA receptor signaling is involved in appetite regulation [59]. Furthermore, these key obesity genes were involved in regulating cation channel activity, synapse maturation, and neurotransmitter receptor activity, which could impact food intake, energy expenditure, and glucose metabolism [60]. Meanwhile, KEGG pathway enrichment analysis revealed that key obesity genes were enriched in signaling pathways in the brain, specifically neurotransmitter signaling, involving dopaminergic, glutamatergic, and cholinergic synapses. Brain scans of humans indicated dopamine-regulated brain circuits were involved in obesity [61], while obese mice on a high-fat diet displayed reduced levels of multiple enzymes involved in dopamine production when switching to the low-fat diet [62]. Changes in glutamate transmission in obese animals showed increased dopamine transmission and altered synaptic functions [63]. Basal forebrain cholinergic signaling was reported to regulate feeding behavior in rats [64], while the frontal cortex and hippocampus displayed functional impairments in cholinergic and synaptic activity, leading to weight gain, hypertension, and dysmetabolism [65]. Moreover, a decrease in growth hormone secretion has been associated with obesity [66] and the suppression of insulin secretion led to weight and fat mass reduction [67]. Our enrichment analysis offers evidence of the complex interplay between key obesity genes and the brain, impacting feeding behavior, energy expenditure, metabolic homeostasis, and insulin secretion.

Hub genes have shown promise as targets for drug development [68]. To validate this hypothesis, we analyzed a recent lipid GWAS [69] and identified hub genes associated with LDL cholesterol from the reported genes. Notably, our analysis revealed the inclusion of *HMGCR* and *PCSK9* as hub genes. These genes have been extensively studied and play crucial roles in hypercholesterolemia treatment. *HMGCR* is a major target of statins, regulating cholesterol levels by inhibiting its expression [70]. Furthermore, *PCSK9* has been linked to blood cholesterol levels, and inhibitors of *PCSK9* have proven effective in lowering LDL cholesterol [71]. These findings further support the potential utilities of hub genes, making them attractive targets for drug development and repurposing.

We examined our key genes with DGI analysis and identified 23 drug-related genes that serve as targets for 78 FDA-approved drugs that showed potential in regulating body weight based on previous studies involving human and animal experiments (Supplementary Table 7). Among these genes, four were targeted by five or more drugs, with *KIT* being the focus of seven weight loss drugs. Among the 47 weight loss-associated drugs, fluoxetine [72] and citalopram [73] are commonly prescribed antidepressants for treating binge eating disorder linked to obesity. Conversely, topiramate [74] and zonisamide [75] are antiepileptic medications capable of suppressing appetite and increase energy expenditure, leading to weight loss. Additionally, metformin [76] has shown effectiveness in promoting weight loss by reducing glucose production in the liver and improving insulin sensitivity. Mesalamine [77] reduces fasting glucose levels and BMI while increasing HDL-cholesterol. Of the 78 candidate drugs listed, SIDER reported 19 drugs with common side effects including asthenia, headache, nausea, fatigue, dermatitis, musculoskeletal discomfort, vomiting, decreased appetite, and diarrhea (Supplementary Fig. 1). While not life-threatening, they can considerably affect the health and well-being of patients, leading to discontinuation of treatment or additional medical attention. We proposed that drugs with high reported side effects (e.g., ribavirin, *n* = 52), may not be suitable for repurposing. Conversely, drugs with low reported side effects, (e.g., duloxetine and paliperidone, *n* = 1; quetiapine, *n* = 2; amisulpride, *n* = 7; oxcarbazepine, *n* = 8), targeting genes with obesity-related knockout abnormalities in mice could be considered for repurposing. However, it is important to note that drug repurposing is a complex process and requires careful evaluation.

Our study has strengths and weaknesses. We translated biological data into functional knowledge and treatment interventions, suggesting promising key obesity genes as targets for new obesity therapeutics. However, the specificity of computational tools and inadequate specific information on biological processes and pathways remained challenging to establish causality. To address this, the integration of multiple credible biological resources and statistical tools could compensate for the specificity limitation of each resource, further enhancing the prioritization of candidate genes and markers [78]. Additionally, our approach explored anti-obesity therapy and uncovered novel repurposed applications and adverse drug reaction information for the key obesity genes. However, we lack knowledge of the interactions between these drugs and the genes in the context of obesity. Furthermore, adverse drug reactions may differ among diverse populations [79]. As a follow-up to our study, we proposed the integration of genome editing techniques, such as CRISPR-Cas9 [80], to validate our prioritized key obesity genes in animal experiments. Supplementing our findings with empirical experiments would improve our comprehension of regulatory gene interactions and their role in obesity, bringing us closer to effective obesity treatment.

## Conclusions

In conclusion, our study provides valuable contributions to the obesity research field by utilizing a systematic data-driven in silico approach to identify and predict novel regulatory genes and potential therapeutic targets for obesity through the translation of GWAS results. Firstly, we prioritized key obesity genes from multiple knowledgebases and identified novel genes which had not been functionally validated in regard to obesity. These genes were involved in various biological pathways, such as lipid and energy metabolism, insulin secretion, adipogenesis, and neural development, adding insights into the underlying mechanism of obesity. Secondly, we identified promising drug-related genes and repurposing drug candidates for novel obesity management. These drugs are capable of regulating energy metabolism and expenditure, appetite control, glucose homeostasis, and insulin sensitivity, offering promising avenues for the development of effective treatments.

### Supplementary information

Supplementary Figure 1

Supplementary Table 1

Supplementary Table 2

Supplementary Table 3

Supplementary Table 4

Supplementary Table 5

Supplementary Table 6

Supplementary Table 7

Supplementary Table 8

Supplementary Table 9

Supplementary Table 10

Supplementary Information

## Data Availability

The codes used in this analysis are available on our GitHub page at https://github.com/angmiayang/integrative_obesity_analysis.git. These codes are freely available, enabling reproducibility and further exploration of our findings.
