# Supplementary material for: Deciphering the genetic landscape of obesity: a data-driven approach to identifying plausible causal genes and therapeutic targets
Source: J Hum Genet. 2023 Aug 24;68(12):823–33. doi: 10.1038/s10038-023-01189-3 (PMC10678330; doi:10.1038/s10038-023-01189-3)
Supplement: Supplementary file 12 — Supplementary Information [file 10038_2023_1189_MOESM12_ESM.docx]

**SUPPLEMENTARY INFORMATION**

Supplementary Figure 1: Heatmap illustrating 19 drug candidates and their corresponding side effects. Out of the total 78 candidates, 19 were reported to have side effects as reported by the SIDER database.

Supplementary Table 1: The detailed predicted regulatory functions of 28,787 obesity-associated SNPs.

Supplementary Table 2: Differentially upregulated obesity genes found in brain tissues at adjusted P <0.05.

Supplementary Table 3: Transcriptionally regulated obesity genes associated with various regions of the brain.

Supplementary Table 4: Network centrality analysis of the prioritized obesity-associated genes is performed using cytoHubba, employing 11 topological analysis methods: Maximal Clique Centrality (MCC), Density of Maximum Neighborhood Component (DMNC), Maximum Neighborhood Component (MNC), Degree, Edge Percolated Component (EPC) and six centralities (Bottleneck, EcCentricity, Closeness, Radiality, Betweenness and Stress) based on shortest paths.

Supplementary Table 5: Enriched GO categories of key obesity genes.

Supplementary Table 6: Enriched signaling pathways of key obesity genes.

Supplementary Table 7: Drug-related genes information of the key obesity genes and their reported weight gain and weight loss information from human and animal experiments.

Supplementary Table 8: Druggable genes information of the key obesity genes.

Supplementary Table 9: Side effect data of the 78 drug candidates.

Supplementary Table 10: Negative and benchmarking analysis.
